# Polymersomes for Sustained Delivery of a Chalcone Derivative Targeting Glioblastoma Cells

**DOI:** 10.3390/brainsci14010082

**Published:** 2024-01-14

**Authors:** Ana Alves, Ana M. Silva, Joana Moreira, Claúdia Nunes, Salette Reis, Madalena Pinto, Honorina Cidade, Francisca Rodrigues, Domingos Ferreira, Paulo C. Costa, Marta Correia-da-Silva

**Affiliations:** 1 UCIBIO—Applied Molecular Biosciences Unit, MedTech-Laboratory of Pharmaceutical Technology, Faculty of Pharmacy, University of Porto, Rua Jorge Viterbo Ferreira 228, 4050-313 Porto, Portugalpccosta@ff.up.pt (P.C.C.); 2 Laboratory of Organic and Pharmaceutical Chemistry, Department of Chemical Sciences, Faculty of Pharmacy, University of Porto, Rua Jorge Viterbo Ferreira 228, 4050-313 Porto, Portugal; 3 Associate Laboratory i4HB—Institute for Health and Bioeconomy, Faculty of Pharmacy, University of Porto, 4050-313 Porto, Portugal; 4 REQUIMTE/LAQV, ISEP, Polytechnic of Porto, Rua Dr. António Bernardino de Almeida, 431, 4200-072 Porto, Portugal; 5 Interdisciplinary Center of Marine and Environment Research (CIIMAR), University of Porto, Terminal dos Cruzeiros do Porto de Leixões, Avenida General Norton de Matos P, 4450-208 Matosinhos, Portugal; 6 LAQV, REQUIMTE—Associated Laboratory for Green Chemistry, Department of Chemical Sciences, Faculty of Pharmacy, University of Porto, Rua Viterbo Ferreira 228, 4050-313 Porto, Portugal

**Keywords:** synthesis, chalcones, glioblastoma, nanotechnology, polymersomes

## Abstract

Glioblastoma (GBM) is a primary malignant tumor of the central nervous system responsible for the most deaths among patients with primary brain tumors. Current therapies for GBM are not effective, with the average survival of GBM patients after diagnosis being limited to a few months. Chemotherapy is difficult in this case due to the heterogeneity of GBM and the high efficacy of the blood–brain barrier, which makes drug absorption into the brain extremely difficult. In a previous study, 3′,4′,3,4,5-trimethoxychalcone (MB) showed antiproliferative and anti-invasion activities toward GBM cells. Polymersomes (PMs) are an attractive, new type of nanoparticle for drug administration, due to their high stability, enhanced circulation time, biodegradability, and sustained drug release. In the present study, different MB formulations, PEG2000-PCL and PEG5000-PCL, were synthesized, characterized, and compared in terms of 14-day stability and in vitro cytotoxicity (hCMEC/D3 and U-373 MG).

## 1. Introduction

More than 10 million cancer cases are reported each year, and cancer is one of the most devastating diseases [1]. Concerning brain cancer [2], approximately 189,000 individuals die annually on a global scale, and glioblastoma (GBM) is the most common and aggressive form of central nervous system tumor [3,4,5,6]. The median overall survival for GBM patients remains around 15 months [5,6]. The prognosis tends to be poor due to some treatment limitations and particularities of this disease, such as being highly invasive and non-localized, having diffuse characteristics, and poorly responding to local drug activity [4,5,6]. The prevalence of this ailment is more commonly observed in males over the age of 45 years than in females and younger ages [7].

The available chemotherapy is not successful due to the blood–brain barrier (BBB) efficacy as well as the heterogeneity of brain cancers [8]. The first step in the treatment of GBM is surgery, followed by radiation and combined therapy with temozolomide (TMZ). TMZ is the standard drug for chemotherapy in GBM [9], reaching “blockbuster” status in 2010 [10], after being approved by the Food and Drug Administration (FDA) in 2005 [11]. TMZ has been already incorporated into liposomes for the treatment of brain tumors [12], with nanocarriers being considered a promising strategy to treat GBM [13]. A new generation of drug delivery system (DDS), namely, polymersomes (PMs), was reported for the first time by Hammer and Discher [14], who described the physical properties of polymeric structures of poly (ethylene oxide)-block-poly (ethylene) di-block copolymers (PEO-b-PEE) that are self-assembled in aqueous environments. PMs are hollow vesicles with an internal environment that is separated from the surrounding aqueous medium by a bilayer of amphiphilic polymers [15]. PMs have emerged as a compelling novel category of nanoparticles in the field of drug delivery owing to their ability to encapsulate both hydrophilic and hydrophobic molecules within their aqueous cavity or hydrophobic membrane. These particles have better physicochemical properties than liposomes, including higher stability, enhanced circulation time, biodegradability, and sustained drug release [14]. Moreover, they can vary in dimensions and charge, and some have been already demonstrated to be biocompatible and biodegradable [16]. PMs can contain chemical groups available on their surface for conjugation with targeting moieties, without compromising their functionality. The anti-cancer drugs doxorubicin and paclitaxel were simultaneously loaded within PMs composed of poly(ethylene glycol)-poly(ε-caprolactone) PEG-PCL and poly(ethylene glycol)-poly-lactic acid PEG-PLA copolymers [17,18]. These PMs delivered these drugs to tumors implanted in mice, and a 50% size reduction in tumors was reported five days after the drug injection. Besides cancer therapy, PMs can be utilized as carriers for gene delivery, enabling the transport of genetic material such as DNA or RNA into cells. The encapsulation of nucleic acids within PMs protects them from degradation and facilitates their efficient uptake into cells, enabling gene therapy applications [19,20].

Natural and synthetic chalcones have attracted the scientific community’s attention due to their broad array of reported biological activities, including antitumor activity, which is produced through the inhibition of diverse molecular targets [21,22,23,24,25]. Our research group has identified several chalcones with notable growth-inhibitory activities in human tumor cell lines [26,27,28]. Among them, 3′,4′,3,4,5-trimethoxychalcone (MB) was found to be one of the most potent in vitro growth inhibitors of several cancer cell lines [27]. In particular, 3′,4′,3,4,5-trimethoxychalcone (MB) displayed potent antiproliferative activity against different cancer cells, and this effect was associated with antimitotic activity [27]. In another work, chalcone MB inhibited the cell metabolic activity of two GBM cell cultures, i.e., human glioblastoma astrocytoma (U87) and murine glioma (GL261), more effectively than other structurally related chalcones [28]. Moreover, the non-tumor endothelial cell line bEnd.3 showed high resistance toward chalcone MB, with a decrease in metabolic activity only at 100 μM, demonstrating some selectivity of MB to GBM cell cultures and not non-tumor cell line bEnd.3 [28]. Another study assessed its ability to reduce the critical hallmark features of GBM and to induce apoptosis and cell cycle arrest and showed that MB successfully reduced the invasion and proliferation capacity of tumor cells, promoting G2/M cell cycle arrest and apoptosis in GBM cell lines [28]. Interestingly, the incorporation of MB into liposomes maintained the inhibitory activity against U 87 [28]. Nevertheless, as liposomes have some disadvantages such as low encapsulation efficiency and poor physical and chemical stability, PMs were developed in this study as alternative DDSs for the inclusion and sustained delivery of this promising anti-GBM compound. For the preparation of PMs, PEG-PCL copolymers were synthesized based on previous research methods published on the preparation of PMs and the encapsulation of anti-cancer drugs such as paclitaxel [29], docetaxel [30], and doxorubicin [31]. After the characterization of the prepared PMs, their cytotoxic effects on the growth of the most representative GBM cell line [32], U-373 MG, and on the growth of a healthy brain endothelial cell line (hCMEC/D3) were evaluated. In this study, free MB was also evaluated for the first time against these two cell lines.

## 2. Materials and Methods

### 2.1. General Information

Chalcone MB was synthesized and characterized by the Laboratory of Organic and Pharmaceutical Chemistry, Department of Chemical Sciences, Faculty of Pharmacy/CIIMAR research group, as previously described [27]. Methoxy PEG 2000, Methoxy PEG 5000, ε-caprolactone, and Sn(oct)_2_ were acquired from Sigma-Aldrich Co. (Sintra, Portugal) and methanol (HPLC grade) from VWR chemicals. IR spectra were obtained in a KBr microplate in an FTIR spectrometer Nicolet iS 10 from Thermo Scientific with the Smart OMNI-Transmission accessory (Software OMNIC 8.3, Thermo Scientific, Madison, WI, USA). ^1^H and ^13^C NMR spectra were recorded in the Department of Chemistry at the University of Aveiro, Portugal, on a Bruker Avance 300 instrument (Bruker Biosciences Corporation, Billerica, MA, USA) (^1^H: 300.13 MHz; ^13^C: 75.47 MHz). ^13^C NMR assignments were made in bidimensional HSQC and HMBC experiments (long-range C, H coupling constants were optimized to 7 and 1 Hz). Chemical shifts are expressed in ppm values relative to tetramethylsilane (TMS) as an internal reference and coupling constants are reported in hertz (Hz). HPLC was performed on a Dionex Ultimate 3000 (Thermo Scientific, Darmstadt, Germany). 

### 2.2. Synthesis and Preparation of Polymersomes

The amphiphilic PEG-PCL diblock copolymer was synthesized via ring-opening polymerization using a microwave-assisted procedure. Briefly, the reaction was carried out under microwave irradiation: firstly, 2.5 g of PEG2000 and methoxyPEG5000 was dried at 120 °C and 1000 w for 10 min; then, 6.55 g ε-Caprolactone (PCL) and 10 μL Sn(oct)_2_ were added to the dried methoxyPEG; the reaction continued at 130 °C for 25 min while stirred at 30 rpm and under 1000 w irradiation. For purification, the synthesized copolymer was dissolved in chloroform and then precipitated by adding an adequate amount of diethyl ether. This procedure was repeated three times and then the precipitate was freeze-dried to remove residual water; following that, the obtained copolymer was kept at −20 °C. The NMR spectrum of PEG-PCL diblock was acquired at room temperature in CDCl_3_.

Self-assembled structures were prepared using the film rehydration method. Briefly, 20 mg of the copolymer and 5 mg of MB in 2 mL of dichloromethane were transferred into a round-bottomed flask. The solvent was evaporated under vacuum using a rotary evaporator. The thin, dried polymer film was hydrated through the addition of 2 mL distilled water at 60 °C and stirred continuously overnight under 1250 rpm. The polymer dispersion was sonicated for 30 min at 25 °C followed by extrusion 20 times through a homogenizer (FPG12800 Pressure Cell Homogeniser, Unit 5 New Horizon Business Center Barrows Road Harlow Essex CM19 5FN UK).

### 2.3. Characterization of Polymersomes

#### 2.3.1. Particle Size and Polydispersion Index

We prepared 40 μL of PMs in 1960 μL purified water and analyzed the samples using dynamic light scattering (DLS) with a ZetaPALS apparatus (Brookhaven Instruments Corporation, Holtsville, NY, USA). The data collected, mean diameter and PDI, through PALS Particle Sizing Software (Version 5, Brookhaven Instruments Corporation, Holtsville, NY, USA) were expressed as mean ± standard deviation.

#### 2.3.2. Thermal Behavior

MB–excipient and excipient–excipient compatibility studies were performed using a DSC 200 F3 Maia (Netzsh–Gerätebau GmbH, Selb, Germany). MB, excipients, and formulations of PMs were weighed directly in DSC aluminum pans and scanned in a range of temperatures of −40 to 340 °C under a nitrogen atmosphere with a 40 mL/min flow. A heating rate of 10 °C/min was used, and the thermograms obtained were observed for any interaction. An empty aluminum pan was used as a reference. The onset and peak maximum temperatures were calculated using Proteus Analysis software (Version 6.1, Netzsh-Gerätebau GmbH, Selb, Germany). The DSC cell was calibrated (sensitivity and temperature) with Hg (m.p. −38.8 °C), In (m.p. 156.6 °C), Sn (m.p. 231.9 °C), Bi (m.p. 271.4 °C), Zn (m.p. 419.5 °C), and CsCl (m.p. 476.0 °C) as standards.

#### 2.3.3. Negative-Staining Transmission Electronic Microscopy

For negative-staining transmission electron microscopy, 10 µL of samples was mounted on Formvar/carbon film-coated mesh nickel grids (Electron Microscopy Sciences, Hatfield, PA, USA) and left standing for 2 min. The liquid in excess was removed with filter paper, and 10 µL of 1% uranyl acetate was added to the grids and left standing for 10 seconds, after which the liquid in excess was removed with filter paper. Visualization was carried out on a JEOL JEM 1400 TEM at 120 kV (Tokyo, Japan). Images were digitally recorded using a CCD digital camera Orious 1100W Tokyo, Japan at the HEMS/i3S of the University of Porto. Transmission electronic microscopy was performed at the HEMS core facility at i3S, University of Porto, Portugal.

#### 2.3.4. Entrapment Efficiency

The obtained formulations were centrifuged (4500 rpm for 15 min) (Model 5804, Eppendorf, Hauppauge, NY, USA). The supernatants were diluted in methanol (1:2) to promote the release of MB encapsulated in PMs. The obtained samples were analysed with HPLC. Chromeleon 7.2 software was used for data acquisition. The chromatographic conditions included a commercially available Acclaim^TM^ 120 C18 (100 × 4.6 mm) column with a particle size of 5 µm from Thermo Fisher Scientific (Bremen, Germany). The optimized mobile phase was water: methanol (25:75, *v*:*v*) following an isocratic flow of 1.0 mL/min for 10 min, and the temperature of the column was set at room temperature. The injection volume was 10 μL, and the detection was performed at 238 nm. A calibration curve for MB was prepared in methanol from five standard solutions: 28 μg/mL, 32 μg/mL, 40 μg/mL, 56 μg/mL and 61 μg/mL. Through interpolation of the calibration curve, the MB concentration in the supernatant was obtained. The theoretical concentration of MB was calculated considering the initial amount of MB added and the dilutions performed throughout the procedure. Thus, the encapsulation efficiency (EE) was calculated as follows:

$$EE\left(\%\right)=\frac{NPC}{TC}\times 100$$

where TC is the theoretical concentration of the MB if the entrapment efficiency is 100% (μg/mL) and NPC is the final concentration of the MB in the nanoparticle (μg/mL).

#### 2.3.5. Stability Study

A stability study was performed for the PM formulations with and without MB. Samples were periodically evaluated regarding mean diameter and PDI at time 0 and after 1, 7 and 14 days at 4 °C.

#### 2.3.6. MTT Cell Viability Assay

Cell reagents were purchased from Gibco (Invitrogen Corporation, Life Technologies, Renfrew, UK). Immortalized human brain capillary endothelial cells (hCMEC/D3 cell line) were kindly donated by Dr. PO Couraud (INSERM, Paris, France). The human astrocytoma U-87 MG cell line was purchased from the American Type Culture Collection (ATCC). The human glioblastoma astrocytoma derived from a malignant tumor (U-373 MG) was obtained from Sigma-Aldrich. For hCMEC/D3, U-87 MG, and U-373 MG, passages 48–49, 53–56, and 16–17 were used, respectively. Cells were cultivated as reported by and Teixeira et al. [33].

For the 3-(4,5-dimethylthiazol-2-yl)-2,5-diphenyltetrazolium bromide (MTT) assay, cells were seeded in 96-well plates (25 × 10^3^ cells/mL) and exposed to different concentrations (0.1, 1, 10, 100 μM) of PEG2000-PCL, PEG5000-PCL, PEG2000-PCL-MB, PEG5000-PCL-MB, and MB for 24 h. Following the removal of the formulations from each well, cells were washed with HBSS. The number of viable cells was determined by adding MTT reagent and incubating for 3 h at 37 °C. DMSO was used to solubilize the crystals. Triton X-100 1% (*w*/*v*) and culture medium were used as negative and positive controls, respectively. The absorbance was read at 590 nm with background subtraction at 630 nm. Results were expressed as percentages of cell viability.

#### 2.3.7. In Vitro Chalcone MB Release

In vitro chalcone MB release studies were performed using a cellulose dialysis bag diffusion technique (Spectra/Por 3 molecular porous membrane tubing) filled with 1 mL of PEG5000-PCL-MB in isotonic phosphate buffer solution (PBS) at pH 6.3. The dialysis membranes were placed in 80 mL of PBS under magnetic stirring at 100 rpm, maintained at 37 °C for 24 h. At fixed time intervals (T0, T1, T2, T3, T4, T5, T24 h), 1 mL of the PBS solutions was withdrawn and the solution obtained was analyzed using HPLC (same conditions described in Section 2.3.4). A calibration curve was prepared from seven standard solutions of MB in methanol (0.02 μg/mL, 0.05 μg/mL, 0.083 μg/mL, 0.12 μg/mL, 0.23 μg/mL, 0.55 μg/mL, and 1.32 μg/mL).

The same procedure was applied to free MB. The studies were performed in triplicate and the cumulative percentage of the released compound was determined by calculating the mean, indicating the standard deviations.

### 2.4. Statistical Analysis

The results are shown as the mean ± standard deviation of three batches of the same formulation (*n* = 3). The results of mean diameter, EE, and cell viability were statistically analyzed using ANOVA, after confirming the normality and homogeneity of the variance with the Shapiro–Wilk and Levene tests. Significance was set at *p* < 0.05. Differences between groups for ANOVA were compared with a post hoc test (Tukey’s HSD), and different letters in the same sample represent significant differences between different concentrations. All the statistical analyses were performed with IBM SPSS Statistics for Windows (Version 28.0., IBM Co., Armonk, NY, USA).

## 3. Results

### 3.1. Synthesis and Characterization of PEG-PCL Diblock Copolymer

**Scheme 1:**
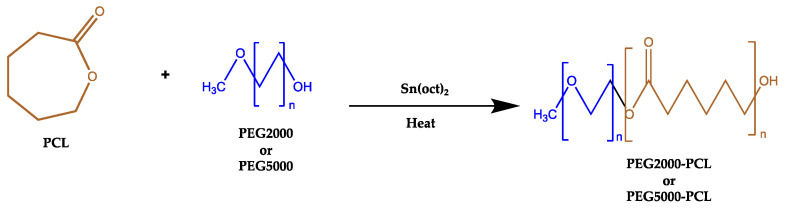
Synthesis of PEG2000-PCL and PEG5000-PCL through ring-opening polymerization. PCL, ε-caprolactone.

The amphiphilic PEG2000-PCL and PEG5000-PCL diblock copolymers were obtained using the ring-opening polymerization (ROP) method (Scheme 1). The most commonly used catalyst, stannous octoate (Sn(Oct)_2_), was selected [34,35]. The macroinitiator, methoxy polyethylene glycol (methoxyPEG2000 and methoxyPEG2000), was dried at 120 °C with microwave irradiation [36] and ε-caprolactone (PCL) was then added [37,38,39].

**Figure 1:**
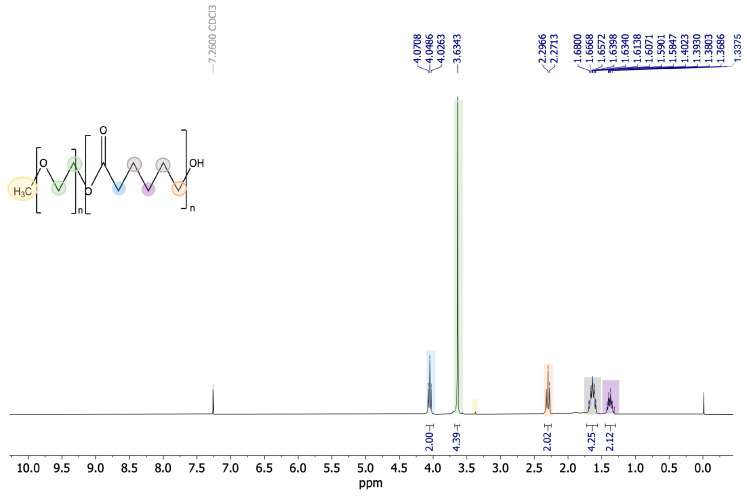
^1^H NMR spectrum (CDCl_3_) of PEG5000-PCL copolymers.

**Figure 2:**
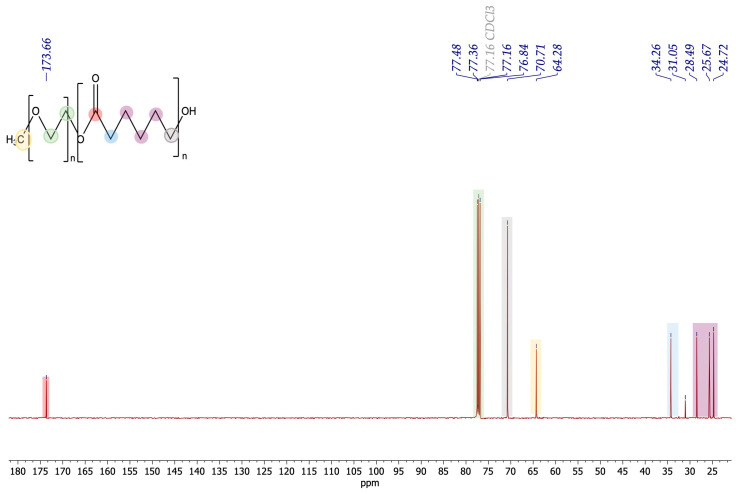
^13^C NMR spectrum (CDCl_3_) of PEG5000-PCL copolymers.

The structure of the diblock copolymers was established using ^1^H and ^13^C NMR (Figure 1), according to Zavvar, T. et al. [40]. The characteristic OCH_2_CH_2_O of the PEG block was assigned to the chemical shift at δ_H_ 3.6 ppm (green, Figure 1). The triplet at δ_H_ 4.1 ppm was assigned to the CH_2_ alpha carbonyl of the PCL block (blue, Figure 1). The -CH_2_ CH_2_ CH_2-_ protons of the PCL block appeared at δ_H_ 1.6 (grey, Figure 1) and 1.4 ppm (purple, Figure 1) as multiplets. Figure 2 shows the ^13^C NMR spectrum of the PEG5000-PCL di-block. Regarding the PEG segment, the aliphatic carbons were detected at δ_C_ 77.4 and 77.3 ppm (green, Figure 2) and the methylene carbon at δ_C_ 64.2 ppm (yellow). Regarding the PCL segment, the signals at δ_C_ 173.6 (red), 76.8 (grey), and 34.2 (blue) ppm were assigned to the carbonyl of the ester -COO-, the carbon linked to the hydroxy, and the CH_2_ alpha carbonyl groups, respectively. The signals of aliphatic carbons were observed at δ_C_ 28.4, 25.6, and 24.7 ppm (pink).

When using Equation (2) to calculate the number of monomers (NMs), we can see that the reaction proceeded successfully, with PEG2000-PCL presenting 45 monomers of PEG2000 and 83 monomers of PCL, while PEG5000-PCL presents 114 monomers of PEG5000 and 108 of PCL. This shows that the polymerization of ε-caprolactone occurred.

NM = (∫CL)/[(Protons of CL/Protons of PEG) × ∫PEG] × Mw PCL

where ∫CL = sum of NMR signals of CL (10.39); Mw PCL = molecular weight of PCL (114 g/mol); Protons of CL = number of theoretical protons of one unit of CL (10); Protons of PEG = number of theoretical protons of one unit of PEG (4); ∫PEG = sum of NMR signals of PEG (4.39).

### 3.2. Particle Size and Polydispersion Index

The mean diameter and polydispersion index (PDI) of PMs are summarized in Table 1. Overall, the mean diameter was less than 200 nm. Empty PEG2000-PCL PMs were larger than PEG5000-PCL PMs, at 128.56 nm and 112.13 nm, respectively. The presence of MB in the PEG5000-PCL and PEG2000-PCL PMs increased the mean diameter of the particles (*p* > 0.05). The ability of particles to effectively travel to the interstitial space through tumor vessel walls depends on the particle size/opening size ratio. In general, the decrease in the particle size improves the transport through tumor vessel walls. A decrease in nanoparticle size is observed with higher molecular weight of the polymer, due to lipophilicity increase in the polymer with the molecular weight [41,42]. Due to the rapid and irregular angiogenesis of tumor tissues, fenestrations and deterioration of blood vessels are common [43,44]. These are open doors for smaller particles; therefore, the smaller the PMs, the greater the possibility of leaking into the tumor interstitial fluid, leading to accumulation and eventually destruction of tumor cells.

The PDI values of 0.1 to 0.3 represent nearly monodisperse preparation, whereas PDI  > 0.4 suggests a broad distribution of macromolecular sizes in solution [45]. For all formulations studied, the PDI was less than or around 0.3, which is generally indicated as a limit for monodisperse preparations.

#### 3.2.1. Thermal Behavior

**Figure 3:**
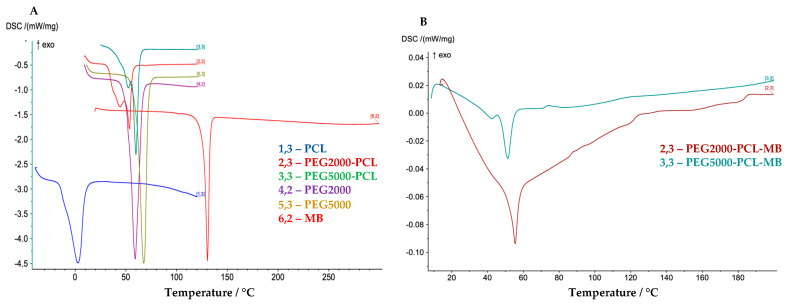
A mixture of empty diblock PMs and their constituents (**A**) and diblock PMs with MB (**B**). PCL—ε-caprolactone; MB—3′,4′,3,4,5-trimethoxychalcone.

DSC thermograms of the PM components are shown in Figure 3A. For the PEG2000 and PEG5000, the onset temperatures were 52 °C and 60 °C and the maximum temperature peaks were 59 °C and 67 °C, respectively. Regarding PCL, the onset was −11 °C and the maximum temperature peak was 3 °C. For the PEG2000-PCL copolymer, the onset and maximum temperatures for the first peak were 32 °C and 44 °C, respectively, and for the second peak were 51 °C and 53 °C, respectively. For PEG5000-PCL, the onset temperatures were 52 °C and 55 °C and the maximum peak temperatures were 52 °C and 59 °C. Here, the different onsets indicate that the crystalline structure was modified for both PCL and PEG. The onset of pure MB was 126 °C and the maximum temperature peak was 130 °C. The PM formulations with MB are shown in Figure 3B. PEG2000-PCL-MB and PEG5000-PCL-MB presented only a peak with the onset, at 48 °C and 53 °C, respectively. These results suggest that MB is molecularly dispersed in the formulations, which can be attested by the absence of its maximum peak. Overall, there was a significant change in the crystalline forms of PEG2000-PCL and PEG5000-PCL with the inclusion of MB.

#### 3.2.2. Negative-Staining Transmission Electron Microscopic Study

The transmission electron microscopy (TEM) technique was used for imaging the PMs prepared with the film rehydration method to evaluate their morphology. 

**Figure 4:**
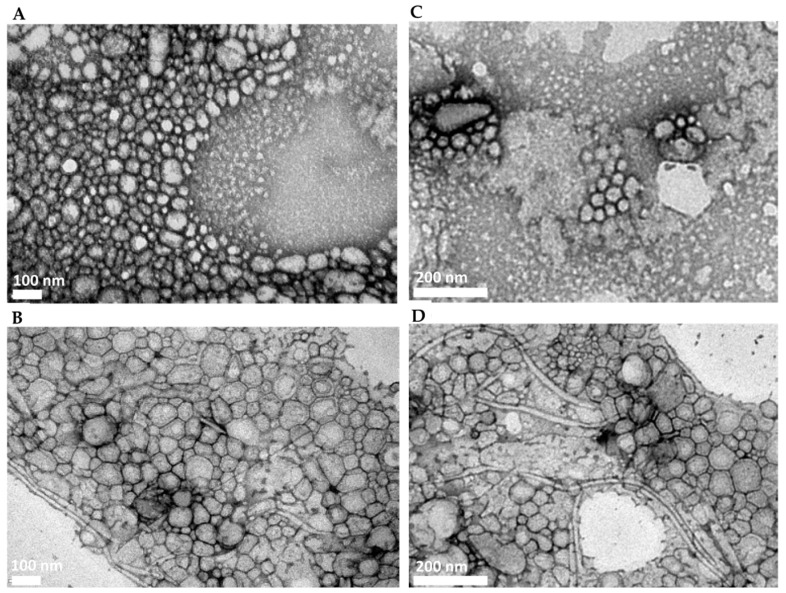
TEM images of PMs formed through film rehydration: (**A**) PEG5000-PCL; (**B**) PEG5000-PCL-MB; (**C**) PEG2000-PCL; (**D**) PEG2000-PCL-MB.

Figure 4A shows a circular shape for the PEG5000-PCL PMs. The size was verified using DLS. In Figure 4B, it is possible to observe that the inclusion of MB does not interfere with the shape or size of the PMs.

Regarding the PEG2000-PCL (Figure 4C) and the PEG2000-PCL-MB (Figure 4D) PMs, the morphology of the particles remains spherical and there is no evidence that MB alters their shape.

#### 3.2.3. Entrapment Efficiency

The entrapment efficiency (EE) of chalcone MB in PMs was determined through indirect measurement of the compound that was encapsulated in the formulation, as conducted via HPLC. Data were fitted to the least squares linear regression, and a calibration curve was obtained: y = 0.0534x − 0.8284. The correlation coefficient was 0.9997, which demonstrates the good linearity in the tested range for MB. Both PMs of PEG5000-PCL-MB and PEG2000-PCL-MB had high EE (98% and 83%, respectively).

#### 3.2.4. Stability Study

**Figure 5:**
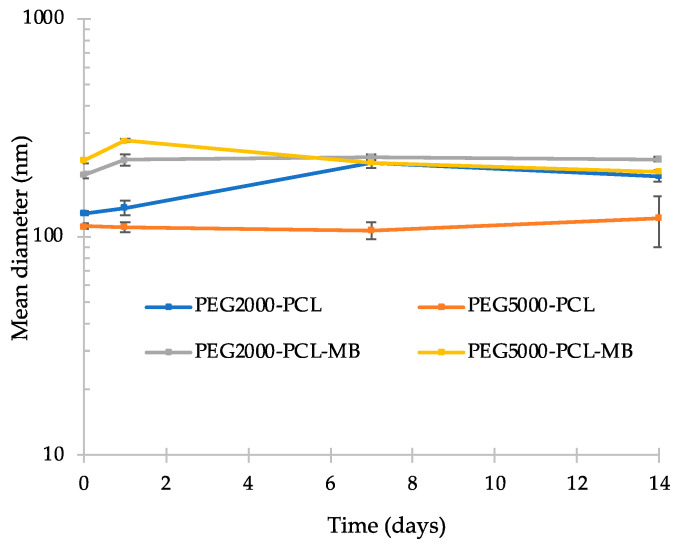
Effective diameter of diblock copolymers PMs, with and without MB, from the day of their production to 1, 7, and 14 days at 4 °C. Values are expressed as mean ± standard deviation (*n* = 3).

The aqueous formulations were stored at 4 °C and showed no relevant change after 1, 7 and 14 days, indicating the stability of the prepared PMs (Figure 5). 

After 1 day, the PEG5000-PCL-MB PMs increased in mean diameter from 223.83 nm to 277.27 nm (*p* = 0.0002), while after 7 days, a mean diameter of 218.87 nm (*p* = 0.0013) was observed. Nevertheless, after 14 days, the particles with and without MB showed sizes of 199.10 nm (*p* = 0.050) and 122.06 nm (*p* = 0.482), respectively. For the PEG5000-PCL PMs, the mean diameter without MB did not show significant differences over time (*p* = 0.444).

Concerning PEG2000-PCL-MB PMs, after 1 day of their preparation, the mean diameter increased from 191.87 nm to 225.77 nm (*p* = 0.014); after 7 days, the formulations showed a mean diameter of 232.37 nm (*p* = 0.478); and after 14 days, the mean diameter was 227.83 nm (*p* = 0.398). PEG2000-PCL PMs without MB did not present significant differences (*p* = 0.323) after 1 day of their preparation. However, after 7 days, the mean diameter increased from 135.83 nm to 218.73 nm (*p* = 0.0006). After 14 days, the particles then showed a mean diameter of 165.87 nm (*p* = 0.155).

#### 3.2.5. Cell Viability Assay

The viability of the glioblastoma U-373 MG cell line and brain endothelial cell line hCMEC/D3 after exposure to PEG2000-PCL and PEG5000-PCL PMs, with and without MB, was evaluated using an MTT assay and compared with the free compound. The U-373 MG cell line was selected for this work since it is a more representative cellular line of the GBM compared to U87 [32].

**Figure 6:**
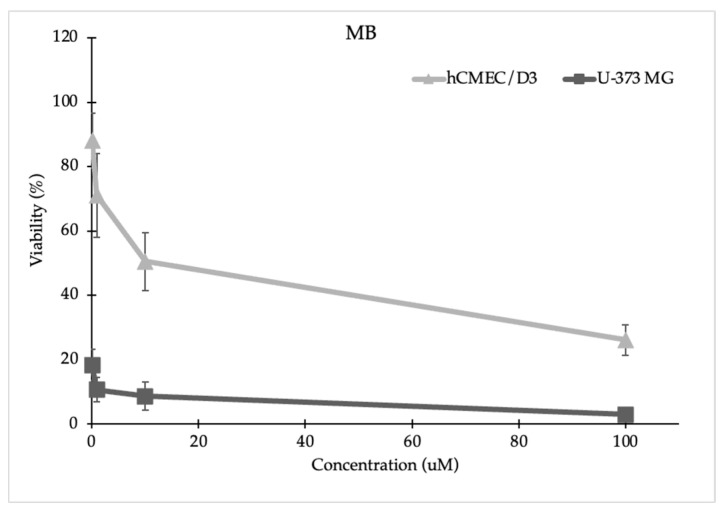
Viability of hCMEC/D3 and U-373MG cell lines (MTT assay) after exposure to different concentrations of MB. Values are expressed as mean ± standard deviation (*n* = 3).

The free compound decreased the viability of the glioblastoma cell line at all tested concentrations (0.1 μM (18.41% ± 4.77), 1 μM (10.70% ± 3.81), 10 μM (8.67% ± 4.37), and 100 μM (3.01% ± 1.91)), while we only observed a decrease in the brain endothelial cell viability at the highest concentrations tested (10 μM (50.54% ± 9.06) and 100 μM (26.15% ± 3.15)) (Figure 6).

**Figure 7:**
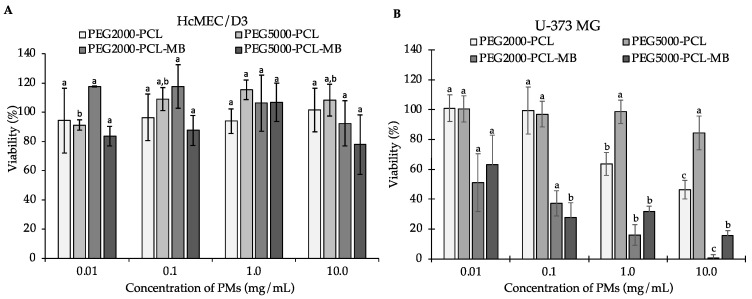
Viability of hCMEC/D3 and U-373MG cell lines (MTT assay) after exposure to different concentrations of PMs with and without MB on the viability of hCMEC/D3 (**A**) and U-373 MG (**B**) cell lines. Values are expressed as mean ± standard deviation (*n* = 3). Different letters (a,b,c) in the same sample represent significant differences (*p* < 0.05) between different concentrations, according to Tukey’s HSD test.

The encapsulation of MB on PMs of PEG2000-PCL and PEG5000-PCL protected the brain endothelial cells from the cytotoxic effect of MB (Figure 7A), while the cell growth of glioblastoma cells decreased significantly at concentrations ≥ 0.01 mg/mL (Figure 7B). The IC_50_ values of PEG2000-PCL-MB and PEG5000-PCL-MB PMs for U-373 MG were 0.093 mg/mL and 0.067 mg/mL, respectively. It is important to highlight that the cell growth of brain endothelial cell lines was not affected in the presence of empty PMs, at any concentration.

Overall, the prepared PMs were able to preserve the viability of the hCMEC/D3 brain endothelial cell lines, which were affected in the presence of free MB, increasing the selectivity for the glioblastoma cell line.

#### 3.2.6. In Vitro Chalcone MB Release

**Figure 8:**
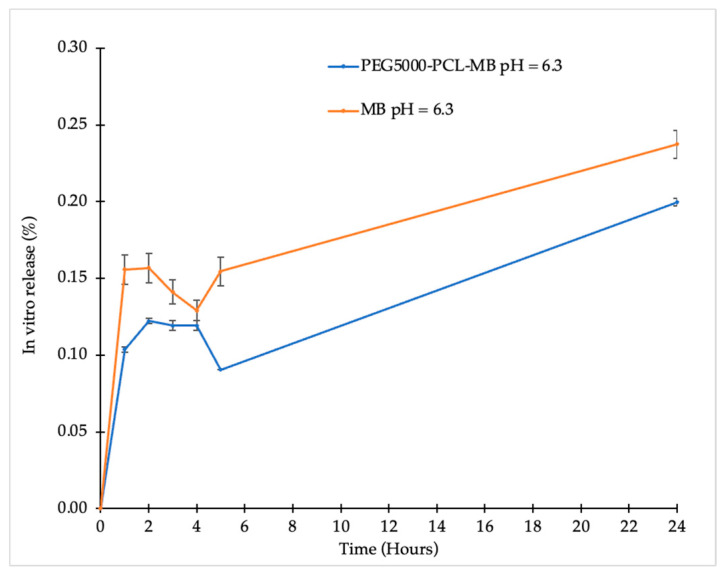
Chalcone MB detected through HPLC in the outer phase of dialysis bags containing free MB or PEG5000-PCL PMs, at pH = 6.3 and 37 °C. Values are expressed as mean ± standard deviation (*n* = 3).

**Figure 9:**
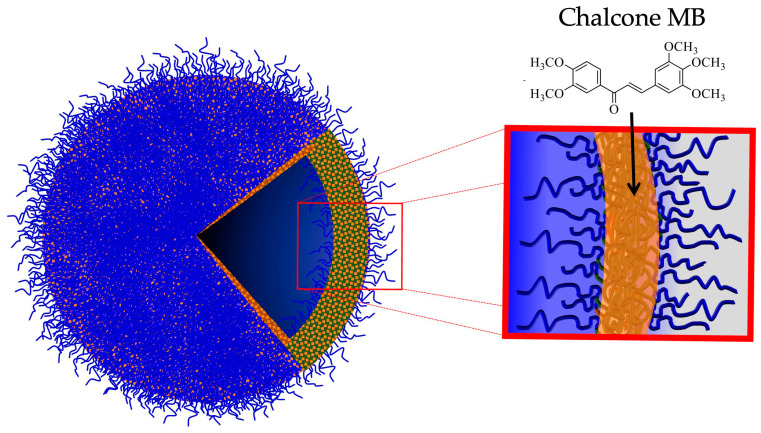
Representation of PM and the chalcone MB.

An in vitro release study of chalcone MB was performed to evaluate the release of the compound from PMs of PEG5000-PCL at pH 6.3 (GBM pHs) over time up to 24 h. Between 0 h and 5 h, slowly increasing amounts of free MB were detected in the outer phase of the membrane bag containing PEG5000-PCL-MB (Figure 8). Moreover, the amount of MB detected in the outer phase of the membrane bag containing PEG5000-PCL-MB was lower than the amount detected when free MB was placed in the bag. The sustained release of lipophilic drugs, such as MB, can be attributed to its entrapment in the hydrophobic part of the PMs (Figure 9) [46]. In vitro studies have shown that drug release depends on the block length of the hydrophobic segment and the crystallinity of the copolymer [47]. PMs have a much slower drug release rate, which is dependent on block length. Certain drugs have been found to release up to 90% after 20 days [47]. Another study showed that the maximum drug release was obtained after a period of 120 h [48].

## 4. Conclusions

In a previous work, the chalcone derivative MB was successfully incorporated into liposomes, while maintaining an inhibitory activity against glioblastoma cell lines. Considering that liposomes have some disadvantages related to low encapsulation efficiency, poor physical and chemical stability, as well as low chemical versatility, more stable particles were obtained in this work—polymersomes (PMs). Diblocks were successfully synthesized via ROP. PEG5000-PCL and PEG2000-PCL PMs showed a mean diameter of about 200 nm. The stability of PMs was tested, and PEG5000-PCL PMs showed no significant changes after 1, 7, and 14 days, while PEG2000-PCL PMs were slightly modified over time. Both formulations presented spherical particles, with a uniform morphology and similar size. Moreover, both formulations exhibited high EE. The chalcone MB displayed cytotoxicity at 10 and 100 µM in the HcMEC/D3 cell line, while the PEG2000-PCL and PEG5000-PCL PMs, with and without MB, did not show any cytotoxicity for this healthy brain endothelial cell line. On the other hand, the PEG2000-PCL-MB and PEG5000-PCL-MB PMs maintained cytotoxicity against the U-373MG glioblastoma cell line at all tested concentrations. Therefore, the prepared PMs with MB were highly selective for the glioblastoma cell line. PMs with the chalcone MB released this compound at pH 6.3; however, a sustained release was observed. This can be attributed to the drug entrapment in the hydrophobic part of the PM. 

Overall, the prepared PMs seem highly attractive nanocarriers for the sustained release of MB. In the future, the crossing of the BBB by these PMs may be studied and, if needed, BBB permeation can be optimized by functionalizing these PMs with transferrin.

## Figures and Tables

**Table 1:** Effective mean diameter and PDI for PM particles of diblock copolymers prepared using the film rehydration method.

Formulation	Effective Mean Diameter (nm)	PDI
PEG2000-PCL	129 ± 2	0.369 ± 0.002
PEG2000-PCL-MB	192 ± 7	0.317 ± 0.010
PEG5000-PCL	112.1 ± 0.9	0.168 ± 0.009
PEG5000-PCL-MB	224 ± 6	0.284 ± 0.001

PEG = methoxy polyethylene glycol; PCL = ε-caprolactone; PDI = polydispersion index. Values are expressed as mean ± standard deviation (*n* = 3).

## Data Availability

Data are contained within this article.
